# Keratin-Alginate Sponges Support Healing of Partial-Thickness Burns

**DOI:** 10.3390/ijms22168594

**Published:** 2021-08-10

**Authors:** Zi Kuang Moay, Luong T. H. Nguyen, Pietradewi Hartrianti, Declan P. Lunny, David Leavesley, Yee Onn Kok, Si Jack Chong, Alvin Wen Choong Chua, Shang-Ian Tee, Kee Woei Ng

**Affiliations:** 1 School of Materials Science and Engineering, Nanyang Technological University, 50 Nanyang Avenue, Singapore 639798, Singapore; zkmoay@ntu.edu.sg; 2 William G. Lowrie Department of Chemical and Biomolecular Engineering, The Ohio State University, 151 West Woodruff, Columbus, OH 43210, USA; nguyen.2032@osu.edu; 3 Department of Pharmacy, School of Life Sciences, Indonesia International Institute for Life Sciences, Jalan Pulomas Barat Kav 88, Jakarta 13210, Indonesia; pietradewi.hartrianti@i3l.ac.id; 4 Skin Research Institute of Singapore—Agency for Science Technology and Research, 11 Mandalay Road, Clinical Sciences Building, Singapore 308232, Singapore; declan.lunny@sris.a-star.edu.sg (D.P.L.); d.leavesley@sris.a-star.edu.sg (D.L.); 5 Department of Plastic Reconstructive & Aesthetic Surgery, Singapore General Hospital, Singapore 169856, Singapore; yeeonn.kok@mohh.com.sg (Y.O.K.); chong_si_jack@hotmail.com (S.J.C.); 6 Division of Musculoskeletal Sciences, Singapore General Hospital, Singapore 169856, Singapore; alvin.chua.w.c@sgh.com.sg; 7 National Skin Centre, 1 Mandalay Road, Singapore 308205, Singapore; sitee@nsc.com.sg; 8 Environmental Chemistry and Materials Centre, Nanyang Environment and Water Research Institute (NEWRI), Nanyang Technological University, 1 Cleantech Loop, CleanTech One, Singapore 637141, Singapore; 9 Department of Environmental Health, Harvard T.H. Chan School of Public Health, 665 Huntington Avenue, Boston, MA 02115, USA

**Keywords:** fibroblast, human hair keratin, sponge, wound healing, porcine burn wounds

## Abstract

Deep partial-thickness burns damage most of the dermis and can cause severe pain, scarring, and mortality if left untreated. This study serves to evaluate the effectiveness of crosslinked keratin–alginate composite sponges as dermal substitutes for deep partial-thickness burns. Crosslinked keratin–alginate sponges were tested for the ability to support human dermal fibroblasts in vitro and to support the closure and healing of partial-thickness burn wounds in *Sus scrofa* pigs. Keratin–alginate composite sponges supported the enhanced proliferation of human dermal fibroblasts compared to alginate-only sponges and exhibited decreased contraction in vitro when compared to keratin only sponges. As dermal substitutes in vivo, the sponges supported the expression of keratin 14, alpha-smooth muscle actin, and collagen IV within wound sites, comparable to collagen sponges. Keratin–alginate composite sponges supported the regeneration of basement membranes in the wounds more than in collagen-treated wounds and non-grafted controls, suggesting the subsequent development of pathological scar tissues may be minimized. Results from this study indicate that crosslinked keratin–alginate sponges are suitable alternative dermal substitutes for clinical applications in wound healing and skin regeneration.

## 1. Introduction

Interest in the use of materials derived from natural sources for clinical applications has been consistently high, primarily due to the rationale that materials derived from natural sources offer better cell and tissue compliance. The processing of natural materials through decellularization and removal of antigenic elements ensures that these materials can be implanted without stimulating significant immune response [1]. Materials such as collagens are used commonly for cell-free and cell-based three-dimensional (3D) dermal templates due to their favorable biocompatibility, processability, and ability to stimulate desirable cell responses [2,3]. This class of materials has also been extensively used as a component in composite materials used for bone implants, patellar tendons, and soft tissue substitutes [4,5]. Hyaluronan is another natural material that is also commonly used for regenerative medicine applications in orthopaedics, cardiovascular medicine, pharmacology, and oncology, due to its ability to facilitate angiogenesis, osteointegration, and cell phenotype preservation [6,7]. These examples hint at the potential for materials from natural sources to be explored for various regenerative medicine applications with better understanding of their innate properties and adapting to them appropriately.

Human hair keratin presents exciting new possibilities to the field of biomedical science as an abundant, bioactive, affordable, sustainable, and potentially autologous source of human-compatible material [8]. In recent years, hair keratin templates in the forms of coatings [9,10], hydrogels [11], sponges [12,13,14], and fibrous mats [15], have been fabricated and demonstrated to support the natural function of living tissues, both in vitro and in vivo. It has been shown that injectable keratin hydrogels were able to increase survival rates in a large animal lethal hemorrhage model, while also exhibiting increased mean arterial pressure and decreased shock index when compared to both commercial chitosan dressing and cotton gauze [16]. In addition, in situ hydrogels fabricated through crosslinking of hair keratins, poly(ethylene glycol) and tyramine were demonstrated to support epithelial-to-mesenchymal transition (EMT) in keratinocytes and facilitate their migration in vitro. These hydrogels also showed enhanced wound-healing capability when tested in a full-thickness excisional wound splinting model in C57BL/6J mice [11]. In another study, keratin films were found to induce hepatocyte attachment via the hepatic asialoglycoprotein receptor (ASGPR), as evident from the absence of the hallmarks of integrin-dependent cell attachment, i.e., focal adhesion formation and the activation of FAK [17]. As the number of studies reporting functional properties of keratin-based templates increases, it is prudent to gain better understanding of these material templates and evaluate them through studies that reflect anatomical and physiological similarities with humans.

Previously, we have demonstrated improved performance of 3D sponges fabricated through covalent crosslinking of keratin and a well-known biomaterial, alginate [13]. Facilitated by 3-(3-dimethylaminopropyl)-1-ethyl-carbodiimide hydrochloride (EDC), the amide bond formation between the two biopolymers has contributed to increased strength and stiffness, resulting in the overall mechanical improvement of the resulting composite material compared to sponges manufactured from keratin alone. More interestingly, the composite sponges were noted to exhibit enhanced bioactivity compared to alginate, which is bioinert. These composite sponges encouraged better tissue ingrowth and neovascularization than existing commercial collagen sponges when measured over a 4-week period in a subcutaneous implant model in mice [13]. Motivated by the encouraging results from the study of subcutaneous implantation in mice, further evaluation of the keratin–alginate sponges was carried out in the current study to demonstrate their efficacy in supporting human dermal fibroblast (HDF) colonization, proliferation, and expression of matrix proteins in vitro. HDFs are known to express α_4_β_1_ integrins while also being critical contributors to cutaneous wound healing and skin regeneration [18,19]. Additionally, a porcine partial-thickness burn wound model was utilized to evaluate the feasibility of these sponges as dermal substitutes.

## 2. Results

In this study, we carried out in-depth characterization of the 3D microarchitecture and structure of the crosslinked keratin–alginate sponges. Scanning electron microscope (SEM) and micro computed tomography (microCT) were used to observe scaffold pore interconnectivity and porosity. Meanwhile, cell compliance was evaluated in vitro using HDF, along with their suitability for in vivo applications, by applying the sponges as substitute dermal scaffolds in a porcine model of deep partial-thickness burns.

### 2.1. Morphology of the Crosslinked Keratin–Alginate Sponge

**Figure 1:**
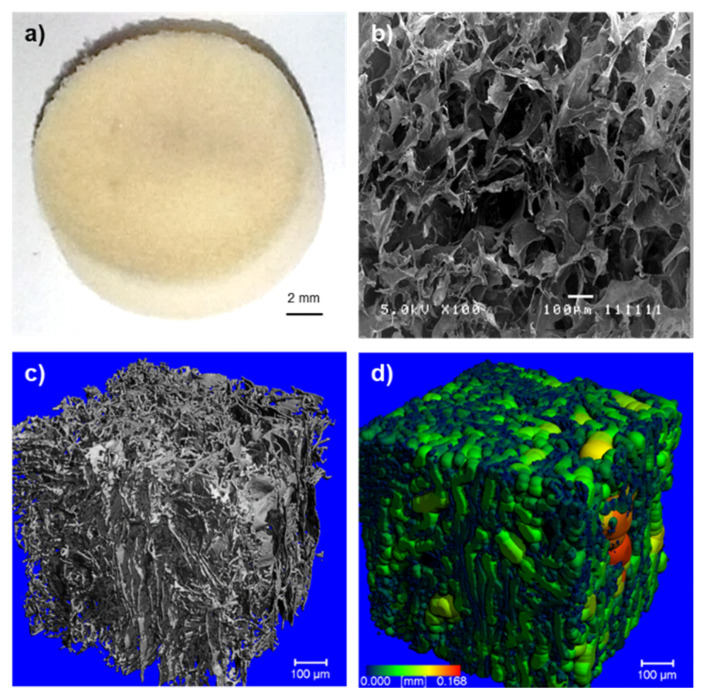
Characterization of the crosslinked keratin–alginate sponge. (**a**) Representative picture of the sponge (scale bar: 2 mm). (**b**) Scanning electron microscopy image of the sponge. (**c**) Three-dimensional (3D) microCT rendering of the sponge and (**d**) the heat map representation of pore diameters within a defined sub-volume.

The crosslinked keratin–alginate sponges were mechanically stable and easy to handle (Figure 1a) [13]. MicroCT images and analysis revealed that the sponges demonstrated a high mean porosity of 93.5 ± 1.0%, a large mean pore diameter of 55.4 ± 4.2 µm, and a high degree of interconnectivity (Figure 1b–d).

### 2.2. Culture of Human Dermal Fibroblasts

**Figure 2:**
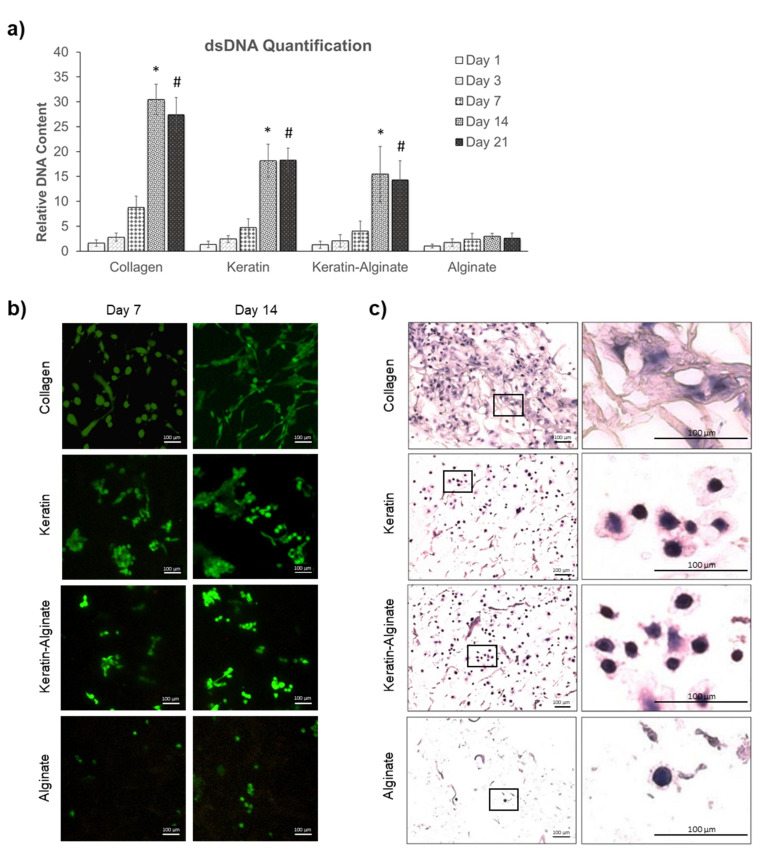
Evaluation of HDF proliferation, viability, morphology, and distribution in the various templates tested. (**a**) Relative amounts of dsDNA over time. Data were normalized against day 1 alginate samples. Each bar represents mean ± SD (*n* = 4). *,^#^ *p* < 0.05 vs. alginate (one-way analysis of variance (ANOVA) and Tukey’s test). (**b**) Live–dead staining images of HDFs cultured in the various templates using the confocal laser microscope. Live cells were stained green (scale bars: 100 µm). (**c**) H&E staining of HDFs after 14 days of culture (scale bars: 100 µm). Rectangles within the left panel images indicate the regions of interest magnified in the right panel.

In vitro cytocompatibility of the crosslinked keratin–alginate sponges was evaluated based on their ability to support the viability, proliferation, and colonization of HDFs (Figure 2). Quantitative measurement of double-stranded DNA (dsDNA) content (Figure 2a) revealed that over 21 days, collagen sponges supported more proliferation of HDFs than any other scaffold tested. In contrast, alginate was a poor substrate for supporting proliferation of HDFs (Figure 2a). Notably, scaffolds made from hair keratin and blended keratin–alginate were also effective in supporting the proliferation of HDF for up to 21 days.

Live–dead staining of colonized sponges revealed that HDFs form small colonies with increased cell densities in all groups from day 7 to 14. Collagen sponges supported more proliferation of HDFs after 7 and 14 days than all other substrates, which is consistent with the dsDNA quantification assay results. Notably, collagen also supported the typical elongated spindle morphology of fibroblasts (Figure 2b). HDFs grown on keratin and keratin–alginate sponges expressed a less contractile morphology, although evidence for cell motility and contraction could be seen in day 14 keratin samples. Consistent with the data from dsDNA assays, alginate sponges offered poor support for HDF proliferation.

Classical hematoxylin and eosin (H&E) staining was used to evaluate the overall cell distribution and anatomy of the scaffolds. It was evident that HDFs were uniformly distributed in collagen, keratin, and keratin–alginate sponges (Figure 2c). At higher magnification, it was evident that HDFs exhibit a more elongated and contractile morphology, which is evidence of firm attachment, when grown on the collagen matrices. On the other hand, HDFs growing on the keratin and keratin–alginate sponges expressed a more rounded morphology, where some exhibited evidence of focal contacts and stress fibers. As expected, alginate sponges were very poor at supporting cell viability and colonization (Figure 2c).

**Figure 3:**
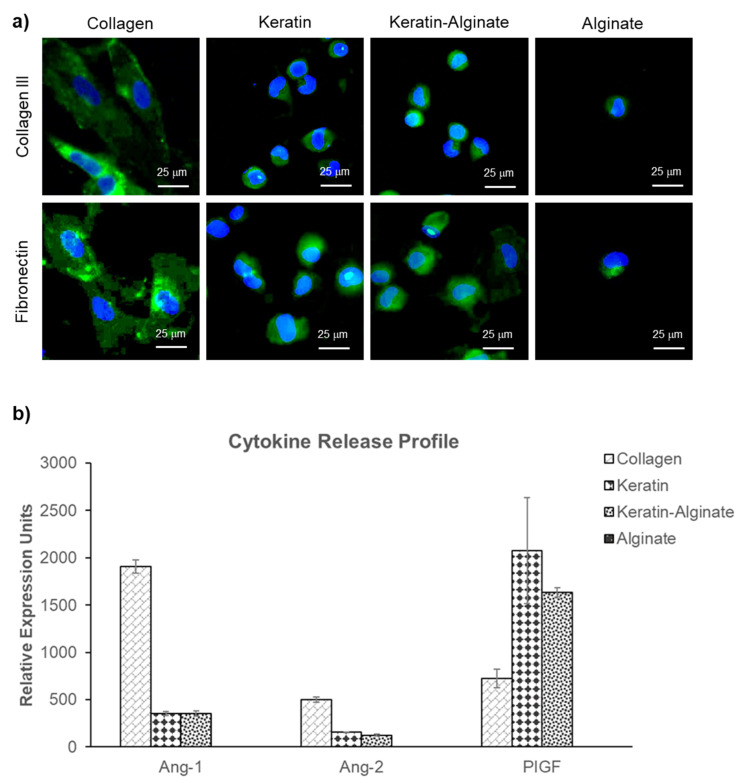
Evaluation of extracellular matrix (ECM) expression and cytokine release of HDFs after culturing for 14 days in collagen, keratin, keratin–alginate, and alginate matrices. (**a**) Immunofluorescence staining of collagen III and fibronectin. Collagen III/fibronectin and cell nuclei were stained green and blue, respectively (scale bar: 25 μm). (**b**) Relative secretion of Ang-1, Ang-2, and PlGF cytokines. Each bar represents mean ± SD (*n* = 2).

In addition, the de novo biosynthesis of extracellular matrix was evaluated, using immunohistochemistry to identify and localize collagen type III and fibronectin (Figure 3a). The analysis demonstrated that collagen type III was produced by HDFs cultured in keratin-containing and alginate sponges, although not as significantly as in the collagen matrices. 

The long-term interests encompass using the crosslinked keratin–alginate sponges for wound-healing applications; thus, it was important to evaluate how dermal cells might respond to the sponges and support wound healing. Analysis of cytokines in conditioned media recovered from HDF cultivated on different sponge materials was done. In particular, cytokines that contribute to angiogenesis were evaluated based on our previous observation that keratin–alginate sponges supported more effective angiogenesis in vivo, such as Angiopoietin-1 (Ang-1), Angiopoietin-2 (Ang-2), and Placenta Growth Factor (PlGF) (Figure 3b) [13]. Predictably, there was no evidence of Ang-1, Ang-2, and PlGF in the conditioned media recovered from HDF cultivated in alginate matrices. HDFs cultured on collagen matrices for 14 days were found to secrete Ang-1 and -2 at five and three times higher, respectively, than those cultured on keratin and keratin–alginate sponges. However, keratin–alginate sponges supported three times more secretion of PlGF compared to that in collagen matrices. Furthermore, collagen, keratin, and keratin–alginate sponges were equivalent when it comes to the secretion of inflammatory mediators (IL-8), thrombogenic factors (TF, uPA, PAI-1), proangiogenic mediators (endostatin, PEGF, IGFBP, TSP-1), and wound repair regulators (TIMP-1) (Supplementary Figure S1).

**Figure 4:**
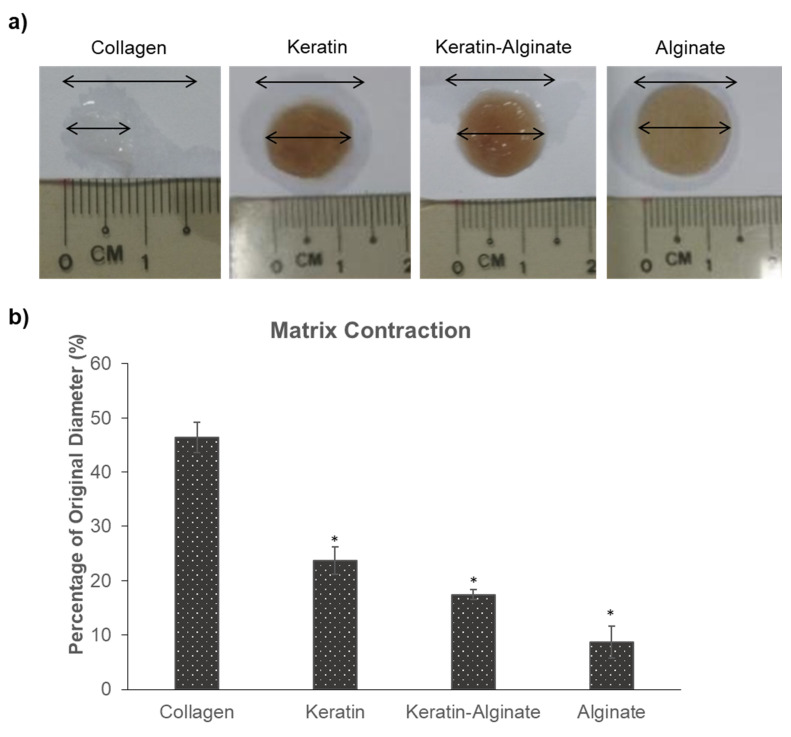
Matrix contraction after 14 days of HDF culture. (**a**) Photographs of matrices after 14 days of culture; Top arrows represent original sponge diameter while bottom arrows represent sponge diameter on day 14 of culture. (**b**) Percentage of scaffold contraction after 14 days of culture (mean ± SD, *n* = 3), * indicates *p* < 0.05 vs. collagen (ANOVA, Tukey’s test).

Graft contraction is one of the important considerations of the suitability of a material as dermal substitutes. Therefore, the change in shape and size of the four types of sponges after 14 days colonization by HDFs was measured. Intriguingly, it was determined that keratin and keratin–alginate sponges displayed less contraction compared to collagen sponges (Figure 4a). Collagen sponges were found to have contracted by 50% of their initial diameter. In contrast, keratin and keratin–alginate sponges contracted significantly less, by 5–20% of their initial dimensions, following 14 days of culture (Figure 4b).

### 2.3. Evaluation of Keratin–Alginate Sponge in Burn Wound Study

Previous studies have evaluated the biocompatibility of the keratin–alginate sponges relative to commercially obtained collagen sponges using mice [13]. However, the mechanisms underlying cutaneous wound healing in rodents is inherently different from the cutaneous wound healing that occurs in primates and humans. It was found that the keratin–alginate sponge exhibited significant cell infiltration and established host vasculature, compared to the same collagen sponges [13]. Herein, the keratin–alginate sponges were also shown to be comparable with the collagen sponges in their ability to induce extracellular matrix (ECM) formation (Supplementary Figures S2 and S3). In the current study, the keratin–alginate sponge was introduced onto a porcine partial-thickness burn wound model to evaluate its effectiveness against that of collagen sponges. For this study, two juvenile male pigs were used, and both animals survived the duration of this study, and healed without any evidence of chronic inflammation or adverse events. Measurements of wound size noted a general decrease of all wounds due to wound healing; there was no statistically significant differences in the wound parameters of the three sample treatments: collagen sponge, keratin–alginate sponge, and standard treatment control (data not shown).

**Figure 5:**
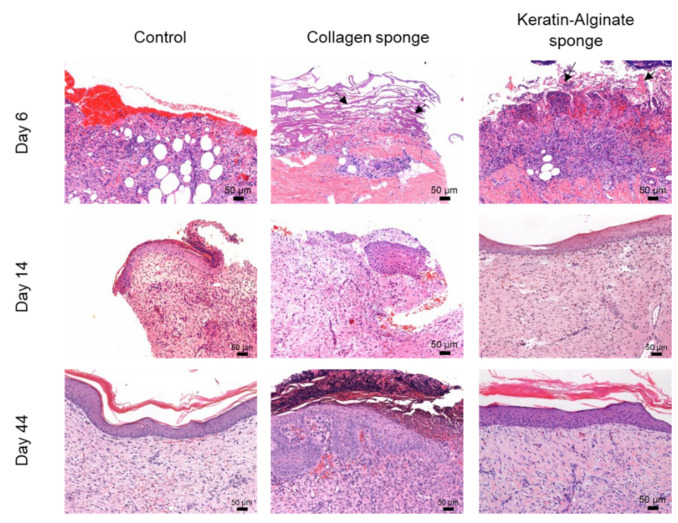
Hematoxylin and eosin evaluation of pig burn wounds treated with collagen and keratin–alginate sponges on days 6, 14, and 44 after grafting. Traces of the collagen and keratin–alginate sponges were evident (black arrows), indicating adequate integration into the host tissue (scale bar: 50 µm).

Evidence for substantial cell infiltration into the grafts was evident within a few days of injury in all sample groups (Figure 5). Wounds treated with the collagen and keratin–alginate sponges did not exhibit thick eschar evident in the control wound. It was noteworthy that the keratin–alginate sponge supported faster re-epithelialization and wound closure than other treatments. Within 14 days post procedure, the epidermis was well-formed beneath the keratin–alginate material. After 44 days, the injury site was phenotypically mature, and there was little evidence of scarring or of residual inflammation.

**Figure 6:**
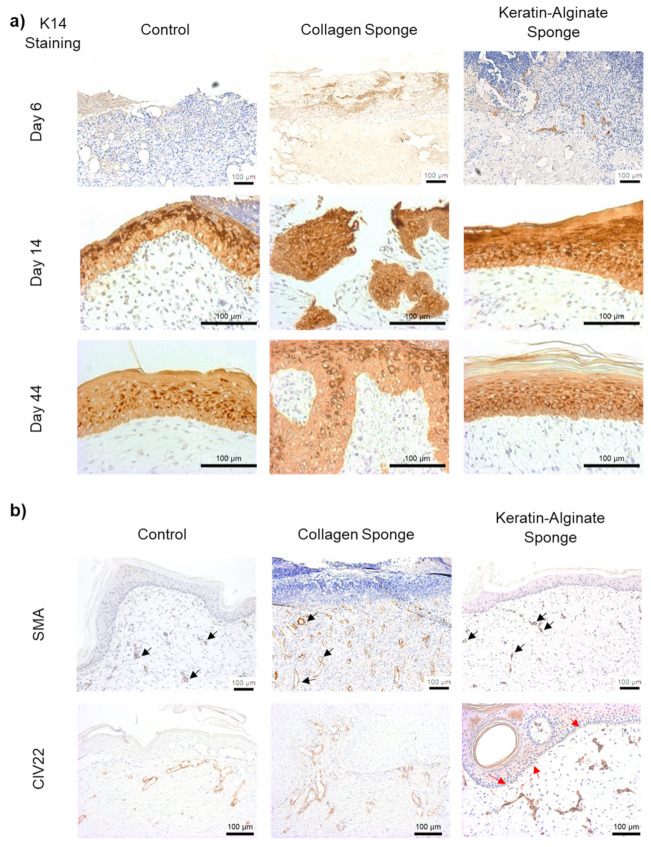
Immunohistochemical staining of wound biopsies. (**a**) Keratin 14 (K14) expression in the sample groups on days 6, 14, and 44. K14 was not found on day 6 due to the debridement of damaged tissue done on day 2 but was expressed at days 14 and 44 as the epidermis regenerates. (**b**) Alpha-smooth muscle actin (SMA) and collagen IV (CIV22) expression in day 44 wounds. Alpha-smooth muscle actin was present in all three groups (black arrows). Collagen IV staining indicate the presence of a mature basement membrane at the epidermal–dermal junction in the keratin–alginate sponge grafted wounds (red arrows), which is not significant in the control and collagen sponge grafted wounds (Scale bar: 100 µm).

The efficacy of re-epithelialization was corroborated by the positive keratin 14 immunohistochemistry results reported in Figure 6a. The lack of an epidermis at day 6 was clear in all three groups due to the absence of keratin 14 staining on the sections, which confirmed the debridement of wounds before the scaffolds were introduced (Figure 6a). Epidermal regeneration was noted on the wound surfaces by day 14 in all three groups, while complete epidermal regeneration was observed by day 44, as seen by a continuous layer of keratin 14-positive cells along the wound surfaces in the three groups. Staining for alpha-smooth muscle actin as well as collagen IV at day 44 revealed that there was increased expression of both markers in the collagen and keratin–alginate groups (Figure 6b). It was noted that both alpha-smooth muscle actin and collagen IV were found in the dermis, particularly in the papillary layer, indicating contraction and the presence of a basement membrane. The stains were also an indication of angiogenesis due to the presence of mature blood vessels in the dermis.

## 3. Discussion

### 3.1. Keratin–Alginate Sponges Support Cell Proliferation and ECM Formation

Biomaterials can interact with cells and induce changes in cell phenotype and response such as cell proliferation, differentiation, and ECM production [20]. It is known that alginate lacks cell-binding motifs, thus making it biologically inactive. Regardless, alginate has worked well as absorbent wound dressings. Common approaches carried out to increase its bioactivity included modification with cell binding peptides such as RGD (Arg-Gly-Asp) via crosslinking [21]. In accordance with this, it was anticipated that the substrates with higher alginate content would display lower cell compliance. Several previous studies have also reported findings that agreed with the result, where alginate did not support the proliferation of several cell types such as fibroblasts and endothelial cells [22,23]. On the other hand, keratin has been revealed to support the growth and adhesion of several cell types such as fibroblasts, hepatocytes, mesenchymal stem cells, endothelial cells, and Schwann cells in both two-dimensional (2D) and 3D environments [9,11,17,23,24]. Twelve of the 17 known human hair keratins contain the LDV (Leu-Asp-Val) cell adhesion motif, which is recognized by α4β1 integrins commonly expressed in some of the cells highlighted earlier [25]. However, keratin alone tends to be mechanically weak; thus, crosslinking with alginate improves stability and handling [13]. This study showed that crosslinked keratin–alginate sponges had the necessary microarchitecture (Figure 1) to support the attachment and proliferation of fibroblasts (Figure 2). 

In terms of ECM production, it was reported that fibroblasts have the tendency to spread anisotropically and the extent of spreading increased with fibronectin density [26]. In the current study, fibronectin expression in the collagen sponges was sufficient, allowing cell adhesion through integrin–ECM binding and resulting in distinctive spreading of fibroblasts (Figure 3a). In contrast, fibronectin was produced in lower amounts within the keratin-based scaffolds, and it was mostly localized in the cytoplasm and surface of the HDFs, possibly resulting in compromised spreading of the cells (Figure 2c). Nonetheless, a previous study suggested that cells cultured on a keratin-coated surface induced more fibronectin production compared to non-coated tissue culture plastic [10]. After 4 weeks of subcutaneous implantation in mice, the keratin–alginate sponges revealed significant cell infiltration with the expression of fibronectin and collagen III at similar intensities compared to collagen sponges (Supplementary Figure S3) [13]. This result suggests that the keratin–alginate sponges are comparable to the commercial collagen sponges in their ability to support ECM formation in vivo.

The contraction observed by the four groups in Figure 4 is a collective outcome of scaffold stiffness, degradation, and the differentiation of fibroblasts into myofibroblasts. Fibroblasts differentiate into myofibroblasts during wound healing, contributing to wound edge contraction and thus accelerating wound closure [27,28,29]. However, extensive wound contraction leads to scarring in humans. It is possible that more fibroblasts have differentiated into myofibroblasts in the collagen matrices in comparison to keratin, alginate, and keratin–alginate sponges, resulting in the higher extent of contraction observed. Keratin, being a cysteine-rich protein, might play a role in inhibiting the expression and activation of matrix metalloproteinase-9 (MMP-9) (Supplementary Figure S1). MMP-9 is a member of the MMP family, which is involved in the degradation of ECM components and an important modulator of wound healing [30]. MMP-9 breaks down ECM components such as collagen to allow the prompt migration of fibroblasts to the wound site, thereby encouraging faster tissue contraction and wound closure [31]. The suppression of MMP-9 activity in keratin-based matrices is an intriguing finding that could be attributed to the “cysteine switch” phenomenon—the downregulation of MMP-9 expression and activity in a cysteine-rich environment [32]. While keratin and keratin–alginate sponges did not perform as well as collagen sponges in supporting cell growth in vitro, they contracted significantly less as well, indicating that keratin-based matrices were more mechanically stable, which could translate into lesser scarring in wound healing.

### 3.2. Angiogenic Ability of Keratin–Alginate Sponges

In the proliferation stage of wound healing, infiltrated monocytes differentiate into macrophages, which secrete and release multiple growth factors and cytokines to promote angiogenesis and stimulate fibroblast migration [33]. Endothelial cells are recruited to the wound site and actively participate in angiogenesis. Ang-1 and Ang-2 are ligands for the Tie-2 receptor [34]. Ang-1 is an agonist for Tie-2, which induces vascular tubule formation, migration, and the survival of endothelial cells. Ang-1 also has a role in angiogenesis by promoting the structural integrity of blood vessels in vivo. Conversely, Ang-2 is a Tie-2 antagonist and interferes with Ang-1-induced vascular stabilization, resulting in an increase in the sensitivity of vessels to other proangiogenic factors. PlGF is a member of the vascular endothelial growth factor (VEGF) family and a multitasking cytokine capable of stimulating angiogenesis by binding and activating VEGFR-1 receptor. The cytokine results revealed that collagen, keratin, and keratin–alginate matrices were comparable in terms of potential to support angiogenesis, while alginate sponges did not support this process (Figure 3b). In the animal study, it was noted that both keratin–alginate and collagen groups supported the significant expression of alpha-smooth muscle actin and collagen IV that showed a distribution pattern hinting at the presence of mature blood vessels in the dermis by day 44 as compared to the control group, indicating possible vascularization (Figure 6b). Data from our previous mouse study also revealed established host neovasculature throughout the keratin–alginate sponges (Supplementary Figure S2).

### 3.3. Epidermis Formation on Keratin–Alginate Sponges

Re-epithelialization occurs during the proliferative phase, and this happens via the migration of keratinocytes from the wound edges and from viable skin appendages in the dermis into the wound area. A basement membrane will form between the dermis and epidermis after re-epithelialization is complete. The basement membrane is important, as it anchors the epidermis for mechanical stability and forms a communication interface between the dermal fibroblasts and epidermal keratinocytes [35,36]. Altered basement membranes are commonly found in scar tissues, which means that the balance of proliferation could be disrupted, promoting the formation of scar tissue. Images of wounds taken at day 44 show that keratin–alginate groups have visually less scarring as compared to the other two groups (Supplementary Figure S4). This result suggests that the keratin–alginate sponges perform as well as if not better than commercial collagen sponges in terms of basement membrane formation and scarring.

## 4. Materials and Methods

### 4.1. Sponge Fabrication

Keratin was extracted from human hair in reducing conditions, as previously reported [24]. Rat tail collagen (type 1) and alginate (alginic acid sodium salt from brown algae) were purchased from BD Biosciences, Franklin Lakes, NJ, USA and Sigma-Aldrich, Burlington, MA, USA, respectively. The fabrication of collagen, alginate, and crosslinked keratin–alginate sponges followed methods described by Hartrianti et al. [13]. For the keratin–alginate sponges, briefly, the carboxylic acid groups of alginates were activated by mixing 1-ethyl-3-(3-dimethylaminopropyl)carbodiimide hydrochloride (EDC) (Chem-Impex International, Wood Dale, IL, USA) with alginate in a 0.15:1 ratio in deionized (DI) water with magnetic stirring for 1 hr at room temperature. Subsequently, freeze-dried keratin powder extracted from human hair was dissolved in DI water and then added into alginate–EDC mixture with a keratin to alginate ratio of 2:1 [13]. The final mixture was stirred for 24 h at room temperature to facilitate complete crosslinking; residual and unreacted EDC was removed by dialysis against DI water using a dialysis membrane with a 10 kDa molecular weight cut-off. Keratin solutions and reagents were sterile filtered before use, and all procedures were performed inside a Class II biosafety cabinet to ensure sterility. Finally, 1 mL of the crosslinked mixture was cast into each well in a 24-well plate, frozen at −80 °C for 24 h, and freeze-dried for 1 day to yield a dry sponge. Larger sponge scaffolds were prepared for the animal study; 85 mL of the mixture was cast in 8.5 cm diameter petri dishes (SPD Scientific, Singapore, Singapore), frozen overnight, and lyophilized per standard protocols. The final product was sterilized by 15 min of exposure to 30 Gy gamma irradiation (Biobeam 8000, Gamma-Service Medical GmbH, Sachsen, Germany).

### 4.2. Scanning Electron Microscopy Analysis

The microarchitecture of the samples was examined with Scanning Electron Microscope (SEM; JEOL 5410) using secondary electron imaging (SEI) mode. Prior to being imaged, samples were snap-frozen in liquid nitrogen and cut to expose their cross-sections. Samples were mounted onto sample holders using carbon tape and sputter-coated with gold at 18 mA for 15 s. SEM images were recorded at an accelerating voltage of 5 kV and spot size of 8 nm.

### 4.3. MicroCT Analysis

The keratin–alginate sponges were analysed using micro computed tomography (microCT) (µCT 50, SCANCO Medical AG, Brüttisellen, Switzerland). The microCT operated with a cone beam originating from a 4 µm focal-spot X-ray tube. Photons were detected using an area-based charge-coupled device (CCD) detector and rendered as a 3400 × 3400 image matrix. Physical parameters were calculated using the maximum fitted spheres method [37].

### 4.4. Human Dermal Fibroblast Culture

Primary HDFs (ATCC, USA) were cultivated in Dulbecco’s Modified Eagle Medium (DMEM) supplemented with 10% fetal bovine serum (FBS), 2 mM L-glutamine, 1 mM sodium pyruvate, 0.1 nM non-essential amino acids, and 100 U/mL penicillin and streptomycin (GIBCO, Gaithersburg, MA, USA). HDFs were harvested when 90% subconfluent using 0.25% trypsin, 0.9 mM EDTA, and counted using a hemocytometer. Sponges (4 mm in thickness and 16 mm in diameter) were seeded with HDF applied to the top surface at a density of 50,000 cells/cm^3^, and cultured at 37 °C in 5% CO_2_, 95% air; media was changed every 3 days. Evaluation of cell viability and cell proliferation were determined using Live/Dead^®^ staining and Picogreen^®^ assay (Invitrogen, Waltham, MA, USA), respectively, following the manufacturer’s guidelines.

### 4.5. Hematoxylin and Eosin (H&E) Staining

After selected periods, samples were harvested and gently rinsed 3 times with phosphate-buffered saline (PBS), before being fixed in 4% paraformaldehyde in PBS overnight at 4 °C. The samples were dehydrated through a graded series of 70, 80, 90, and 100% ethanol, before clearing in 100% xylene. After clearing, the samples were infiltrated with paraffin wax at 65 °C for two 2-h cycles; 5 µm thick sections were collected on glass slides and processed for staining with hematoxylin and eosin (Thermo Fisher Scientific, Waltham, MA, USA) using the manufacturer’s recommended protocol.

### 4.6. Immunofluorescence Staining of Cultured Sponges

The biosynthesis of ECM by HDFs cultured in the 3D sponges was evaluated using immunohistochemistry. The sections were heated to 65 °C for 30 min, dewaxed in 100% xylene, and rehydrated using graded ethanols of 100, 90, 80, and 70%, followed by water. Rehydrated sections were subjected to heat-induced epitope retrieval by heating to 120 °C in Dako Target Retrieval Solution (pH 6, Agilent, Santa Clara, CA, USA) in a pressure cooker (2100 Retriever, Aptum Biologics Ltd., Southampton, UK) [38]. Slides were cooled under running tap water and re-equilibrated to PBS, before being blocked with 1% bovine serum albumin (BSA) in PBS containing 0.05% Tween 20 (Sigma-Aldrich, Burlington, MA, USA; PBST) for 1 hr at room temperature. Thereafter, individual samples were incubated with primary antibodies recognizing collagen III (ab7778, rabbit polyclonal against human collagen III; Abcam, Cambridge, UK) and fibronectin (ab2413, rabbit polyclonal against human fibronectin; Abcam, Cambridge, UK), diluted 200-fold and 250-fold respectively, in blocking solution and incubated at 4 °C, overnight. Negative control samples were similarly processed but without primary antibodies where the blocking solution was left on in place of the primary antibody. Then, the sections were washed 3 times with PBS and incubated for 1 hr in the dark at room temperature with Alexa Fluor™ 488 goat anti-rabbit secondary antibody (Invitrogen, Waltham, MA, USA), diluted 200-fold in the same blocking solution. Finally, sections were washed 3 times with PBS, treated with ProLong Gold Antifade Mountant containing 4′,6-diamidino-2-phenylindole (DAPI) (Invitrogen, Waltham, MA, USA) and overlaid with glass cover slips, and imaged with a fluorescence microscope.

### 4.7. Evaluation of Cytokine Release

The presence of cytokines was assayed using the Proteome Profiler™ Human Angiogenesis Antibody Array from R&D Systems, Minneapolis, MN, USA. Briefly, supernatants from individual samples were recovered after 14 days of culture, centrifuged briefly to remove cell debris, applied to the nitrocellulose array (blocked with buffer supplied by the manufacturer), and incubated at 4 °C overnight, according to the manufacturer’s instructions. Subsequently, each array was washed 3 times with the supplied washing buffer and then incubated with the streptavidin–horseradish peroxidase (HRP) working solution (1:200) for 30 min at room temperature. The arrays were washed 3 times with the washing buffer and placed onto individual plastic sheet protectors. A Chemi reagent mixture (chemiluminescent reagent) was applied onto each membrane, incubated for 1 min, and chemiluminescence was recorded for 2 min using an image analyzer (ImageQuant LAS 4000, GE Life Sciences, Chicago, IL, USA). Images were analyzed for signal intensity using Image Studio™ Acquisition software (LI-COR, Lincoln, NE, USA). Signal intensities were normalized to the cell numbers and used for the direct comparison of cytokine expression levels.

### 4.8. Matrix Contraction

The contraction of the collagen, keratin, keratin–alginate, and alginate sponges were measured manually, using a ruler placed onto the upper surface of each sample. Measures were recorded at the start and after 14 days of culture, and contractions were calculated as percentage change from the original diameter.

### 4.9. Burn Wound Study

The animal study was approved by the SingHealth Institutional Animal Care and Use Committee (IACUC 2016/SHS/1209) and performed under the Guidelines on the Care and Use of Animals for Scientific Purposes developed by the National Advisory Committee for Laboratory Animal Research (NACLAR). Two *Sus scrofa* male pigs (8 and 16 weeks old, weighing 15 kg and 36 kg respectively) were used in the study. Anaesthesia was induced via intramuscular administration of ketamine and diazepam at a dose of 15 mg/kg body weight and maintained with inhalation of 2–3% isoflurane. At day 0, the pigs were shaved, and six 8 cm diameter wounds were created on the flanks of each pig using a modified Pyrex bottle filled with hot water and applied for 40 s to cause a deep partial-thickness burn [39]. The wounds were dressed with Jelonet™ and Melolin™ tulle gras and Opsite (Smith and Nephew, London, UK). After 48 h, necrotic tissue and eschar was removed by excision. Punch biopsies were collected from each wound to verify and evaluate the depth of each burn injury. Following debridement, wounds were assigned to varying treatment arms (Control [*n* = 3; C1–C3], PELNAC™ (referred to as collagen sponge) [Gunze Ltd., Ayabe, Japan] [*n* = 1; P1], keratin–alginate sponge [*n* = 5; K1–K5]). Prior to use, the collagen sponge was immersed in PBS, leaving the protective silicone layer intact. These were applied onto the wounds with the silicone layer facing outwards. Keratin–alginate sponges were placed on the wound bed and dressed with Tegaderm, which were additionally sutured in place. All wounds were dressed externally with Vetrap™ (3M, Maplewood, MN, USA) and non-stick Urgotul (Convatec, Reading, Berkshire, England), tulle gras, Opsite, gamgee, crepe bandage, and a protective garment to prevent the animals from disturbing their dressings. For dressing changes, the animals were sedated with an intramuscular dose of ketamine/xylazine (2.1 mg/kg ketamine, 0.16 mg/kg xylazine). Every wound was inspected and photographed during each external dressing change to record wound parameters. Punch biopsies were collected during these time points from each sample group for histological assessment. The biopsies collected were fixed in 10% formalin and embedded in paraffin. Dressings were changed at days 6, 14, 16, 22, 30, 37, 44, and 51 post-injury. Animals were euthanized on day 51.

### 4.10. Immunohistochemistry Analysis

In order to evaluate re-epithelialization, vascularization, and the formation of granulation tissue, immunohistochemistry was done on formalin-fixed, paraffin-embedded tissue sections acquired from each wound. After deparaffinization and rehydration, samples were subjected to heat-induced epitope retrieval; slides were immersed in Dako Target Retrieval Solution (pH 6, Agilent, Santa Clara, CA, USA) and heated to 120 °C for 20 min in a pressure cooker (2100 Retriever, Aptum Biologics Ltd., Southampton, UK) [38]. Residual peroxidase activity was quenched using Dako REAL™ Peroxidase-Blocking Solution (Agilent-Dako, Santa Clara, CA, USA) for 30 min at room temperature. Samples analyzed for collagen IV were additionally pre-treated with proteinase K solution for 5 min. Non-specific antibody binding was minimized by treatment for 30 min at room temperature with 10% goat serum in PBS. Tissue sections were incubated for 2 hrs with target-specific primary antibodies diluted in blocking solution: (1) mouse monoclonal against keratin 14, LL001 (neat), Santa Cruz Biotechnology, Dallas, Texas, USA; (2) rabbit polyclonal against pig alpha-smooth muscle actin, ab5694 (diluted 1/50), Abcam, Cambridge, UK; (3) mouse monoclonal against pig collagen IV, clone CIV-22 (diluted 1/50), Agilent-Dako, Santa Clara, CA, USA. Samples were washed under running tap water for 10 min and twice with PBST for 5 min each. Appropriate species-specific secondary antibodies (EnVision FLEX DAB+ Substrate Chromogen System, Agilent-Dako, Santa Clara, CA, USA) were hybridized for 30 min at room temperature; and antibody-binding localized with EnVision FLEX DAB+ Substrate Chromogen System (Agilent-Dako, Santa Clara, CA, USA). Samples were counterstained with hematoxylin, dehydrated with ethanol and cleared in xylene, cover-slipped, and mounted with Cytoseal^TM^ Mounting Medium (Thermo Scientific, Waltham, MA, USA). Images were captured using an Olympus IX53 microscope (Olympus, Tokyo, Japan).

### 4.11. Statistical Analysis

Quantitative results are represented as mean ± standard deviation. Comparisons of means were carried out using ANOVA and Tukey’s post hoc analysis. Statistical significance was accepted when *p* < 0.05.

## 5. Conclusions

As proven in the previous study, the presence of alginate in the keratin–alginate sponges helped to increase the tensile strength, tensile modulus, and compression modulus compared to keratin alone [9]. In this study, compared to collagen sponges, keratin–alginate sponges were found to be superior in terms of neovascularization and basement membrane formation. While there was no fibrotic encapsulation around the keratin–alginate sponges, keratin hydrogels have been shown to be encapsulated by thin fibrotic capsules in subcutaneous implantations, suggesting the suitability of keratin sponges over keratin hydrogels in host tissue integration [11,40]. Taken collectively, it is found that the keratin–alginate sponges are potentially suitable for clinical exploitation as dermal substitutes.

## Data Availability

The data presented in this study are available on request from the corresponding author due to privacy.

## Supplementary Materials

The following are available online at https://www.mdpi.com/article/10.3390/ijms22168594/s1.
