# Supplementary material for: Keratin-Alginate Sponges Support Healing of Partial-Thickness Burns
Source: Int J Mol Sci. 2021 Aug 10;22(16):8594. doi: 10.3390/ijms22168594 (PMC8395243; doi:10.3390/ijms22168594)
Supplement: Supplementary file 1 [file ijms-22-08594-s001.zip › ijms-1321419-supplementary.pdf]

# Keratin-Alginate Sponges Support Healing of Partial-Thickness Burns

## SUPPLEMENTARY INFORMATION

## MATERIALS AND METHODS

**Subcutaneous Implantation**. The animal study was performed in compliance with guidelines set forth by the National Advisory Committee for Laboratory Animal Research (NACLAR) and approved by Nanyang Technological University - Institutional Animal Care and Use Committee (NTU-IACUC). Male 6-week-old C57BL/6NTac mice with approximate weight of 20 – 22 g each were obtained from InVivos Ptd. Ltd., Singapore. The mice were anesthetised by intraperitoneal administration of ketamine/xylazine at a dosage of 100 – 200 mg $kg^{-1}$ body weight. After shaving, the skin on the backs of the animals was wiped with 70 % ethanol and a subcutaneous pouch of 10 × 10 mm was made to place each implant. Each animal received bilateral implants including the keratin-alginate sponge and a positive control, PELNAC™ (Gunze Limited, Japan), which is made of porcine tendon-derived atelocollagen. These implants had the same diameter of 8 mm diameter × 3 mm thickness. After implantation, the wound was covered with a Tegaderm™ film (3M Medical, USA). At predetermined time points (weeks 2 and 4), six mice were sacrificed using carbon dioxide. The implants were then harvested together with the skin layer, fixed in 4 % paraformaldehyde, and embedded in paraffin. Sections cut at a thickness of 5 μm were deparaffinised and processed using standard Hematoxylin and Eosin (H&E) and Masson's Trichrome staining protocols to examine general tissue morphology.

**Immunoperoxidase Staining in Mouse Study**. In order to evaluate ECM formation, material degradation, and inflammation response immunoperoxidase staining was performed on the paraffin sections. After deparaffinisation and rehydration, the immunoreactivity of the samples was recovered using heat-induced epitope retrieval method, in which the slides were immersed in Dako Target

Retrieval Solution (pH 6 or 9, Agilent, USA) in a pressure cooker (2100 Retriever, Aptum Biologics Ltd., UK). Dako REAL™ Peroxidase-Blocking Solution (Agilent, USA) was then applied for 30 min at room temperature to quench endogenous peroxidase activity. To prevent the nonspecific binding of antibodies, the samples were blocked with 10 % goat serum in phosphate-buffered saline (PBS) for 1 hr at room temperature. Four primary antibodies against collagen III (ab7778, Rabbit polyclonal to Collagen III; Abcam, UK), fibronectin (ab23750, Rabbit polyclonal to Fibronectin; Abcam, UK), hair cortex cytokeratin (ab197395, Rabbit polyclonal to hair cortex Cytokeratin; Abcam, UK), and CD68 (ab125212, Rabbit polyclonal to CD68; Abcam, UK) were diluted 100-, 600-, 100-, and 1000-fold, respectively, in the blocking solution and incubated with the mouse samples overnight at 4 $^{o}C$ in a humidified chamber. After washing once with running tap water for 10 min and twice with PBS containing 0.05 % Tween 20 (Sigma Aldrich, USA; PBST) for 5 min each, a secondary antibody (EnVision™+/HRP anti-rabbit, Agilent, USA) was applied onto the samples for 30 min at room temperature, followed by $DAB^{+}$ (Agilent, USA) substrate development for 30 s – 5 min. The reaction with $DAB^{+}$ was stopped by immersing the slides in tap water once brown colour was observed. Subsequently, the samples were counterstained with hematoxylin, dehydrated with ethanol and xylene, and mounted with Cytoseal$^{TM}$ Mounting Medium (Thermo Scientific, USA). Images at various magnifications were captured using an inverted optical microscope (IX53, Olympus, Japan).

**RESULTS**

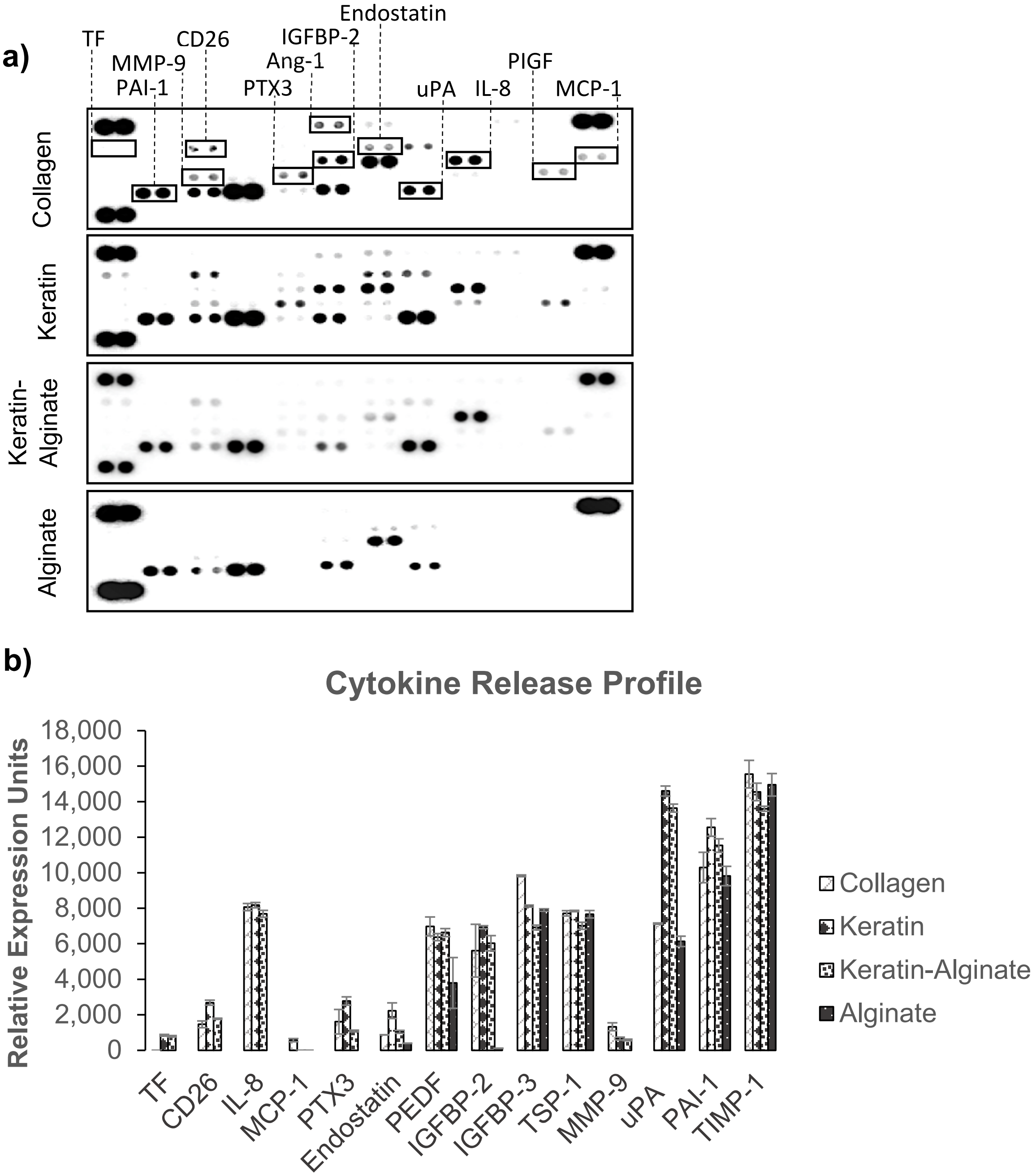


**Figure S1.** Cytokine release evaluation of collagen, keratin, keratin-alginate, and alginate sponges. a) Chemiluminescent images of cytokine array results from HDFs cultured on various sponges at day 14 post-seeding. Cytokines of significance differences in expression levels are labeled in the first image. b) Relative expression of growth factors, cytokines, and enzymes secreted by HDFs after culturing for 14 days on collagen, keratin, keratin-alginate, and alginate matrices. Each bar represents mean ± SD (n = 2). Cytokines used are Tissue Factor (TF), CD26, Interleukin-8 (IL-8), Monocyte chemoattractant protein (MCP-1), Pentraxin 3 (PTX3), Endostatin, Pigment epithelium-derived factor (PEDF), Insulin-like growth factor binding proteins (IGFBP-2 and -3), Thrombospondin-1 (TSP-1), Matrix metalloproteinase-9 (MMP-9), Urokinase Plasminogen Activator (uPA), Plasminogen Activator Inhibitor-1 (PAI-1), and Tissue inhibitor of metalloproteinase-1 (TIMP-1).

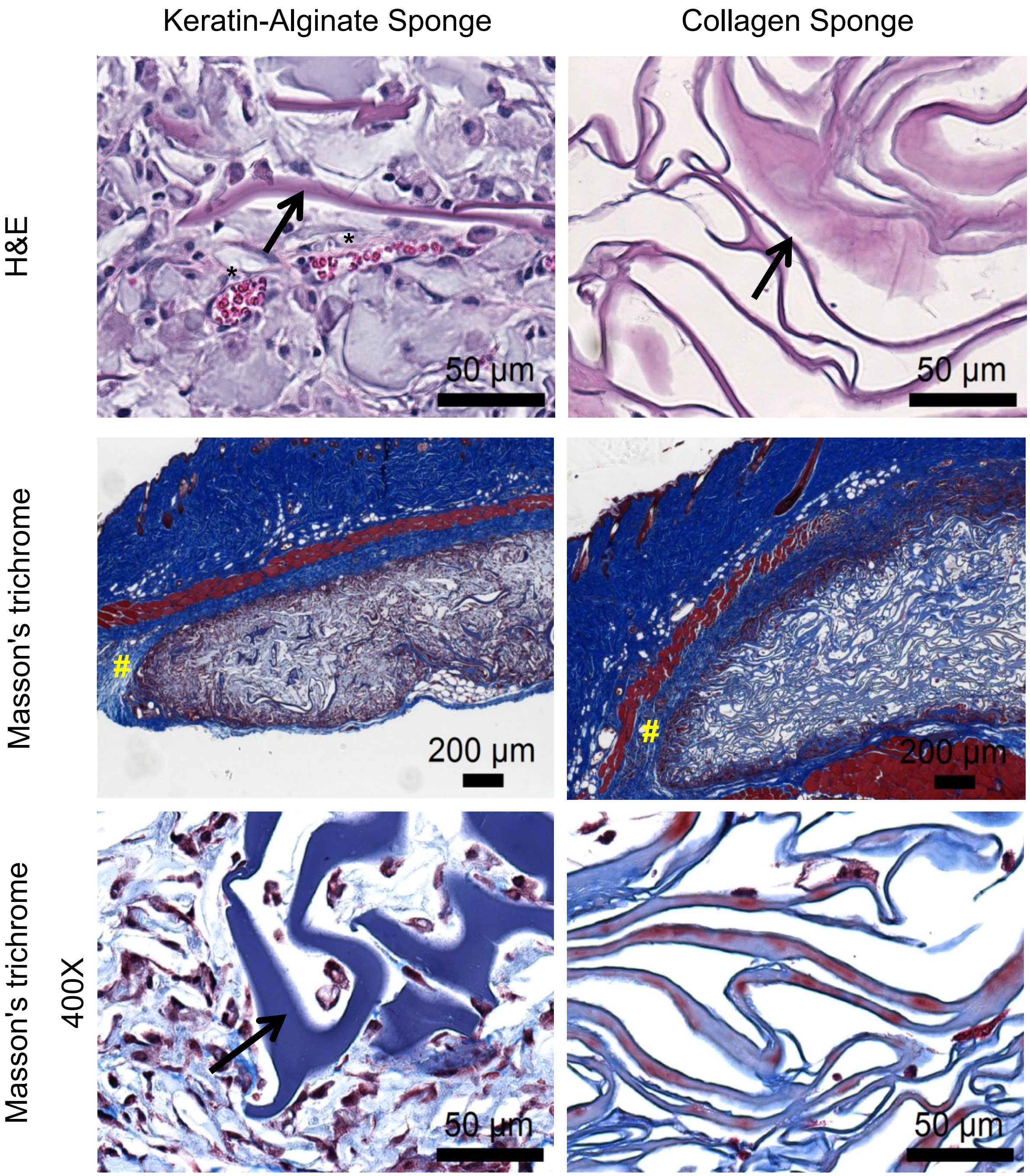


**Figure S2.** Hematoxylin and eosin and Masson's trichrome staining images of the keratin-alginate and collagen (PELNAC$^{TM}$) implants harvested after 2-week implantation in C57BL/6NTac mice. Arrows indicate traces of the implanted material. Neovascularisation could be observed in the keratin-alginate sponge, as shown by red blood cells occupying the capillary lumen (*). Matrix remodelling was more prominent in the collagen sponges, especially along the implant surfaces (#).

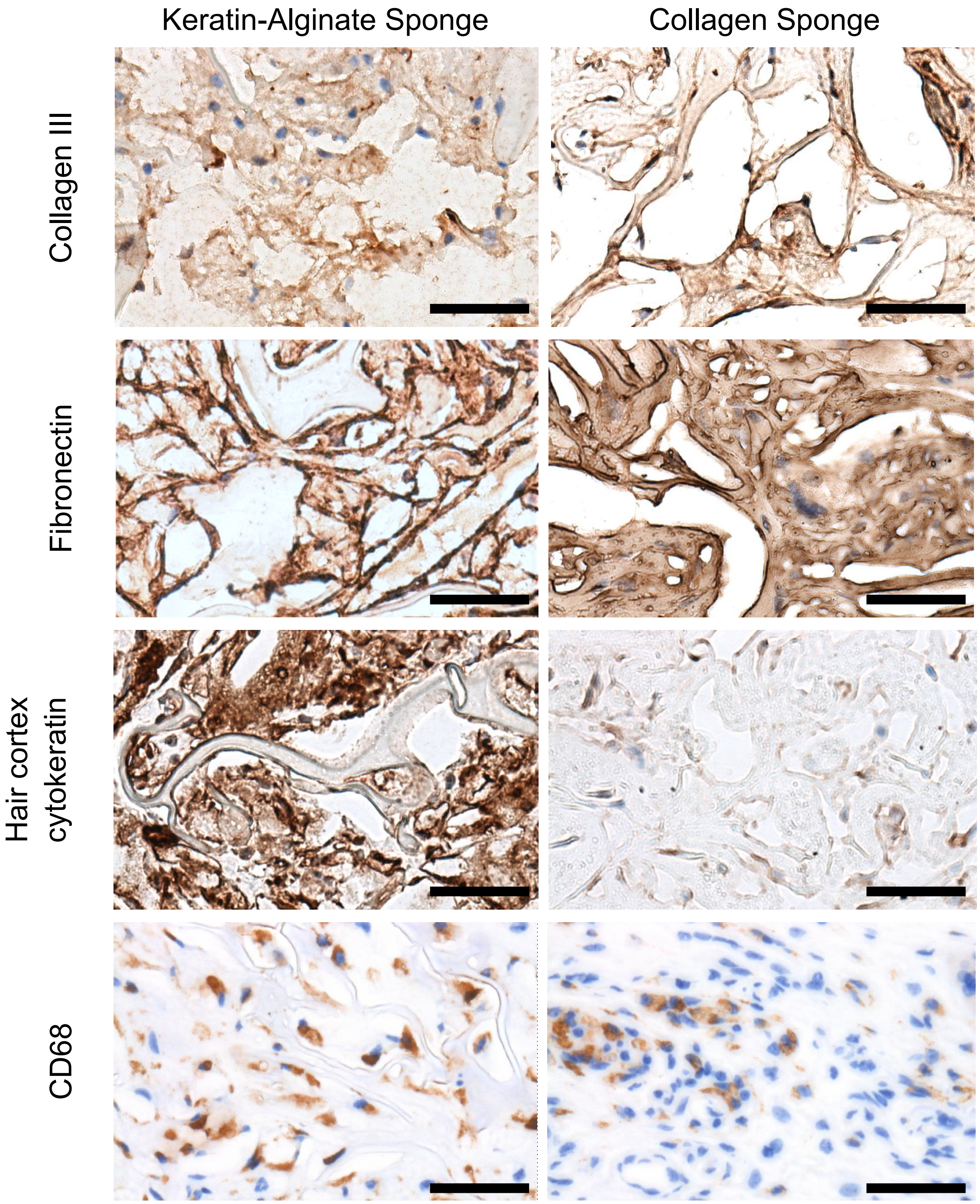


**Figure S3.** Immunohistochemical staining images of keratin-alginate and collagen (PELNAC) sponges harvested after 4-week implantation in C57BL/6NTac mice. Scale bars: 50μm. Comparable deposition of extracellular matrix proteins (collagen III and fibronectin) between these two implants was observed. After 4 weeks, the keratin-alginate sponge was still present in the implantation site as shown by hair cortex keratin staining. Compared to the collagen implants, the keratin-alginate sponges revealed higher density of macrophages (CD68-positive cells).

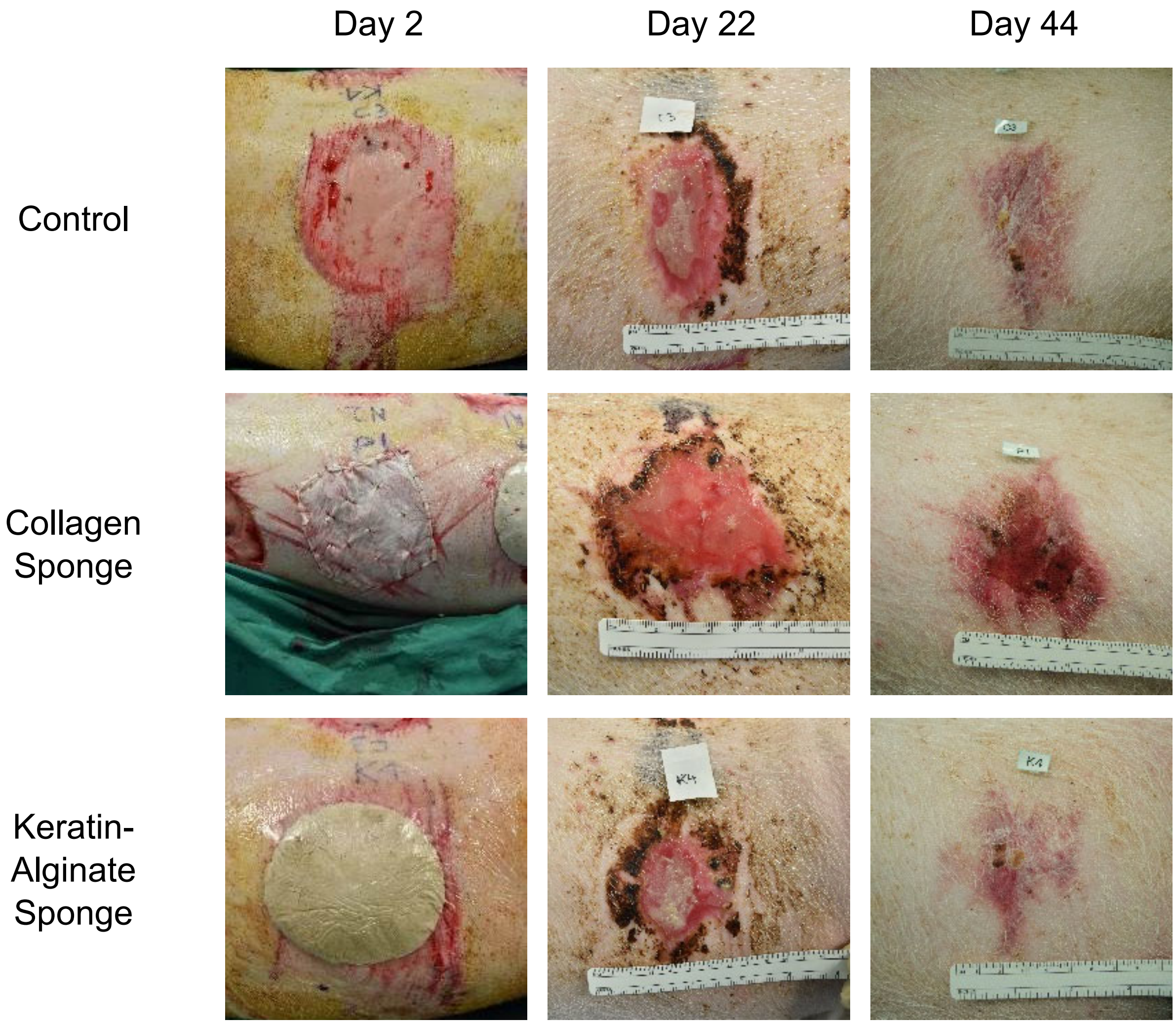


**Figure S4.** Images taken of the wounds during the pig burn wound study on day 2, 22, and 44 respectively. Silicone layer of collagen (PELNAC™) sponge was removed by day 22, which allowed for better imaging of the wounds. All groups had reduced wound sizes by day 44.
